# How, why, for whom, and in what contexts organisational and team dynamics shape, and are shaped by, service user researchers in higher education mental health research centres: a realist review protocol

**DOI:** 10.1186/s40900-026-00980-8

**Published:** 2026-09-23

**Authors:** Alejandro Argüelles Bullón, Fiona Lobban, Andrew Harding

**Affiliations:** Division of Health Research, Faculty of Health and Medicine, Lancaster University, Furness Building, Hazelrigg Ln, Lancaster, LA1 4YG UK

**Keywords:** Service user involvement, Lived experience research, Patient and public involvement, Mental health research, Higher education institutions, Organisational culture, Interdisciplinary research teams, Realist review, Programme theory

## Abstract

**Background:**

Service user researchers (SURs) are increasingly recognised as integral contributors within academic mental health research centres. However, the ways in which these roles are integrated and supported vary widely across academic contexts and are strongly shaped by organisational structures, team cultures and role clarity. While some SURs can contribute meaningfully and thrive, others experience challenges such as emotional labour and limited structural support. There remains a lack of theory-driven understanding of how organisational and team dynamics shape, and are shaped by, the SUR experiences and contributions. This protocol outlines the methods and approach for an ongoing realist review exploring how, why, for whom, and in what contexts organisational and team dynamics within academic contexts shape, and are shaped by, the experiences and contributions of SURs.

**Methods:**

Realist approaches aim to identify the underlying mechanisms that produce outcomes for different groups in different contexts. This approach is well suited to examining the complex and context-sensitive dynamics of SURs roles within academic mental health research teams. This realist review is organised into five phases. The first phase, now completed, involved defining the scope of the review and developing initial programme theories through consultation with key stakeholders. Subsequent phases will include systematic searching of academic and grey literature, quality appraisal, data extraction, realist analysis and synthesis, conducted iteratively with ongoing stakeholder engagement.

**Discussion:**

By making explicit the mechanisms and contextual conditions underpinning SUR roles and organisational and team dynamics, this realist review aims to generate evidence-based recommendations to strengthen organisational and team dynamics in academic contexts. Identifying how specific organisational practices and cultural norms shape and are shaped by SUR experiences and contributions will inform how these roles can be more effectively designed and embedded across diverse academic contexts. Findings from this review will inform a subsequent phase of realist interviews with key informants, supporting further iterative theory refinement.

**Systematic review registration:**

PROSPERO CRD420261296394.

**Supplementary information:**

The online version contains supplementary material available at https://doi.org/10.1186/s40900-026-00980-8.

## Abstract (plain language summary)

People with lived experience of mental health difficulties are increasingly being employed by universities as researchers. These roles can bring important knowledge, challenge assumptions and improve the relevance of research. However, they are not always well supported or valued. Experiences can vary depending on the academic team, the university environment and how the role is understood and organised. Some lived experience researchers can contribute meaningfully and feel respected as part of the team. Others may receive little support or find that their knowledge is not taken seriously. We still need to understand more about why these differences happen and how academic organisations both shape, and are shaped by, the experiences and contributions of lived experience researchers. This study aims to explore how, why, and in what contexts academic team dynamics help or prevent lived experience researchers from thriving in universities. It uses a realist approach, which is useful for understanding complex situations involving different people, roles, relationships, and organisational structures. The study is organised into five phases. The first phase has been completed and involved working with key partners to develop an initial explanation of the issue. Later phases will involve searching the literature, assessing the quality of included studies, and extracting and analysing data to refine the initial explanation. Public involvement will continue throughout the study. By developing a clearer explanation of these issues, this work aims to produce recommendations that can help universities strengthen team dynamics and better support the experiences and contributions of lived experience researchers.

**Supplementary information:**

The online version contains supplementary material available at https://doi.org/10.1186/s40900-026-00980-8.

## Background

Within academic settings, service user researchers (SURs) are designated roles that seek to meaningfully embed lived experience in the design, conduct and dissemination of mental health research [1, 2]. Unlike academic researchers whose primary expertise is derived from conventional academic training and career pathways, SURs are specifically appointed on the basis of their lived experience of mental health difficulties and their ability to draw upon this experiential knowledge within research processes. While some SURs may also hold academic qualifications or research training, their role is distinguished by the explicit recognition and mobilisation of lived experience as a form of expertise within academic research environments [3, 4]. The establishment of SUR roles has gained momentum in the UK and has become increasingly common across Higher Education Mental Health Research Centres (HEMHRCs), defined in this work as universities or other higher education institutions that conduct mental health research and involve SURs in the research process. This development has been driven both by growing recognition of the value of lived experience perspectives in research and by funder requirements or journal guidelines mandating lived experience involvement [5, 6]. For example, the National Institute of Health Research (NIHR) provides guidance outlining good practice for working with SURs [7]. This guidance specifies expectations for involving lived experience across the research lifecycle, including setting the research agenda and priorities, guidance on planning equitable and meaningful partnerships, teaching on the importance of involving people with lived experience and how to evaluate and report impact. HEMHRCs, such as the Service User Research Enterprise (SURE) at King’s College London, represent further institutional efforts to embed SUR roles within academia by creating dedicated organisational structures and integrating lived-experience expertise into research design, delivery and governance [8]. Similar approaches have also been increasingly adopted internationally in both high and low-middle income countries with varying degrees of success [1, 9–11]. Taken together, this growth and recognition of SUR roles highlight the importance of understanding how their contributions and experiences can be most effectively integrated within HEMHRCs.

Widespread employment of SURs is a recent development, and there is considerable variation in how effectively they are embedded across institutions [12]. A range of terms, including “peer researcher,” “survivor researcher,” and “expert by experience,” are used to describe these roles [13]. In the context of this study, SURs will be defined as individuals whose role explicitly requires mental health lived experience, who work within HEMHRCs and who are formally affiliated with a university or university-led research project through a contractual arrangement. Although differing labels do not necessarily denote different functions, they often signal variations in institutional intent and levels of role integration. Some degree of variation is expected, and may be appropriate, depending on the needs of specific projects. However, this diversity, alongside the absence of shared frameworks or consistent role definitions, has contributed to a landscape in which SURs’ responsibilities, employment conditions and perceived legitimacy vary widely [14, 15]. SURs operate within complex institutional systems and must navigate multiple relationships across research, human resources and governance teams. They do not work in a vacuum, but within dynamic contexts that shape the interpretation and impact of their contributions [16–18]. Consequently, the experiences and contributions of SURs within academic settings have been uneven. While some have been meaningfully involved in shaping research, others have encountered marginalisation or unclear role boundaries [19–21]. These challenges are frequently compounded by broader issues of epistemic legitimacy, structural inequality and power dynamics, which may affect SURs’ experiences and contributions, and in turn influence the quality of research processes and organisational dynamics [22, 23].

Against this backdrop, the organisational and team dynamics of HEMHRCs represent a particularly important context for examining SUR roles. Academic research environments are shaped by distinctive institutional logics, including bureaucratic processes, competitive funding structures, performance metrics, and hierarchies of expertise, all of which influence how knowledge is produced and valued [24–26]. Within HEMHRCs, SURs operate at the intersection of research teams, administrative systems and governance structures, requiring them to navigate complex relationships across disciplinary and epistemic boundaries [22, 27]. These settings are also characterised by entrenched knowledge systems and power asymmetries that can reproduce epistemic injustices, while simultaneously offering opportunities to challenge dominant assumptions through lived experience expertise [28–30]. This also raises a more fundamental question about what is valued and why involvement is pursued, as early clarity and shared understanding of purpose are central to sustaining meaningful involvement and avoiding downstream challenges [31–33].

At the individual level, SURs may experience tokenistic involvement, stigmatising interactions or distress when engaging with sensitive experiences, which can cause pain and anxiety. However, these impacts are not consistently reported, and the available evidence often reflects researchers’ perceptions rather than SURs’ own accounts [34–36]. At the team level, SURs may encounter role ambiguity, emotional labour, and ongoing identity negotiation, shaped by colleagues’ expectations and institutional norms [37, 38]. At the organisational level, limited time, resources, and understanding of lived experience involvement can further constrain meaningful integration [39–41]. These dynamics make HEMHRCs a critical area of study, as they can either enable SURs to thrive and exert influence, or require them to expend significant effort simply to survive within academic systems.

In summary, the literature points to several organisational and team dynamics that influence whether SURs can thrive or merely survive within HEMHRCs, including the clarity and legitimacy of SUR roles, the distribution of power and epistemic authority within research teams and the availability of organisational support, time, and resources. Across this literature, effective integration and alignment between SURs, teams and broader organisational structures emerge as vital conditions shaping both SURs’ experiences and the impact of their contributions to the wider academic organisation. Examining these organisational and team processes therefore offers valuable insight into the conditions under which SUR contributions are supported or constrained within contemporary mental health research.

From this, it becomes evident that SURs operate within multilayered and interrelated contexts in which they may face substantial challenges. When attention is diverted towards navigating organisational demands, and when their contributions and experiences are diluted or not taken seriously, SUR roles risk becoming symbolic or virtue-signalling exercises rather than meaningful contributions to mental health research. Existing research has explored aspects of SURs’ experiences and contributions within academic settings, as well as barriers and facilitators [42–46] however, much of this work has relied on more traditional positivist or constructivist approaches. While valuable, these method-driven approaches do not fully address what it is about academic contexts that enables SURs to survive or thrive. To date, existing knowledge has not yet been synthesised into a theory-driven explanation to HEMHRCs and SUR roles. In response, this review aims to develop a programme theory to guide the development of SUR roles that both enable meaningful contributions and support positive experiences, while also providing HEMHRCs with evidence-informed recommendations for engaging and working sustainably with SURs. Developing an evidence-based, theory-informed understanding of how academic organisations implement SUR roles is therefore essential to minimise unintended negative consequences and to support meaningful lived experience involvement in mental health research.

The underlying mechanisms (defined as context-sensitive, hidden causal forces) and the ways in which they interact with differing contextual conditions [47, 48] to enable SURs to thrive or merely survive have not yet been theorised specifically in this context. This work adopts a theory-driven approach that asks not only what works, but for whom, how, and in what contexts [49, 50] and will address these gaps by developing a programme theory of how organisational and team dynamics within HEMHRCs shape, and are shaped by, the experiences and contributions of SURs. The findings will support more intentional, equitable and impactful integration of lived experience roles within mental health research settings. As the realist approach continues to evolve, this protocol also aims to enhance transparency in the review process and provide practical insight for others seeking to understand how realist reviews are conducted in practice.

### Aim

The aim of this protocol is to outline the approach and methods for a realist review that will explore how organisational and team dynamics within HEMHRCs shape, and are shaped by, the experiences and contributions of SURs, and how these dynamics interact with contextual conditions and generative mechanisms to produce intended and unintended outcomes.

### Research questions

1. How, why, for whom, and in what contexts do organisational and team dynamics within HEMHRCs shape, and are shaped by, the experiences and contributions of SURs?
2. What context-sensitive mechanisms are triggered by the employment of SURs in HEMHRCs, and how do these mechanisms both shape SURs’ experiences and contributions and influence academic cultures and power relations within HEMHRCs?
3. What programme theories explain how SUR roles are intended to function within HEMHRCs, and how do these theories enable or constrain SURs’ ability to thrive, survive or influence change across different academic settings?

### Registration of protocol

The protocol was registered on 30.01.2026 on PROSPERO (CRD420261296394).

## Methods/Design

This study adopts a realist approach, which is well suited to investigating complex interventions and social phenomena. Rather than asking solely whether something works, realist research focuses on how, why, for whom, and under what circumstances outcomes are generated (or not) [49, 51]. Causation is typically explained using the context–mechanism–outcome (CMO) heuristic, or related configurational approaches, to capture generative explanations of how outcomes arise in specific contexts [52]. Unlike traditional, method-driven systematic reviews that prioritise effectiveness, realist reviews employ a theory-driven and iterative approach to evidence synthesis, drawing on both academic and grey literature [53]. A realist review seeks to open the “black box” of causation by examining the underlying mechanisms that explain how and why outcomes occur within particular contexts, rather than assessing effectiveness at a surface level [47].

The review will be guided by an iterative five-step process, conducted in line with RAMESES publication standards for realist synthesis: (1) refining the scope and developing an initial programme theory (IPT); (2) searching for evidence; (3) selecting and appraising studies; (4) data extraction; (5) synthesis and refinement of programme theories through analysis and stakeholder consultation [54–56] and the PRISMA-P guidelines (see Additional File 1). Although these steps are presented sequentially, the review process will be iterative, with stages revisited, conducted in parallel, or repeated as required in response to emerging findings and theoretical development.

**Step 1: Refining the scope and developing an initial programme theory**

Before commencing the review, a key step in any realist project is to ensure that key concepts and definitions are clearly articulated and agreed upon by the research team. This is particularly important given the existence of multiple, and sometimes competing, definitions of realist concepts within the literature [52, 57]. Through team discussions and consideration of alternative definitions, the definitions presented in Table 1 were selected as most appropriate for the aims of this review, with accompanying justifications.

**Table 1:** Definitions of core realist concepts and outcomes applied in this review

Key term	Working definition	Reference	Justification
Context	*The dynamic, relational and layered conditions (social, institutional, temporal and material) that interact with mechanisms to influence outcomes.*	Greenhalgh and Manzano [48]	Context in realist evaluation and synthesis has often received less attention than mechanisms and is sometimes mischaracterised as comprising only static, observable features. Greenhalgh and Manzano’s [48] conceptualisation instead emphasises the complexity and interactivity of contextual conditions.
Mechanism	*A context-sensitive, hidden causal force*	Astbury and Leeuw [47]; Pawson and Tilley [58]	The conceptualisations proposed by Astbury and Leeuw [47] and Pawson and Tilley [58] are adopted because they encompass the three key characteristics of mechanisms from a realist perspective: they are hidden, causal, and context sensitive. Keeping the definition deliberately open allows mechanisms to be understood more broadly.
Outcome	*The intended or unintended consequences of a particular intervention, which can be proximal or distal*	Pawson and Tilley [49]	Recognising that outcomes may be both intended and unintended broadens the understanding of “what works, for whom, and in what contexts,” rather than focusing solely on whether an intervention appears to work. Considering proximal and distal outcomes also strengthens the theoretical logic by tracing the causal chain through which mechanisms generate immediate (proximal) effects that may, in turn, contribute to longer-term (distal) outcomes.
Context-Mechanism-Outcome Configuration (CMOC)	*Heuristic used to explain generative causation, helping to reflect on the relationship between a context, mechanism and an outcome of interest.*	De Weger et al. [52]	The original CMO configuration is retained. This review does not focus on a specific programme or intervention, but rather on understanding why the dynamics of the academic context may or may not trigger mechanisms that contribute to SUR engagement and experiences and vice versa. To avoid complicating the core explanatory form of the CMO, actors will instead be discussed more broadly in relation to the question of “for whom?” rather than being embedded directly within the configuration.
Programme theory	*An explanation of how and why an intervention is expected to work, often expressed as a CMOC*	Coleman et al. [59]	In this review, each CMOC is treated as an individual programme theory, which is then progressively refined to develop an overarching middle-range theory.
Middle range theory	*Clearly formulated explanatory frameworks that operate at a level of abstraction sufficient to identify and generalise causal mechanisms across different contexts while still being grounded in empirical observations*	Merton [60]; Mukumbang & Wong [61]; Pawson [62].	A central concept in realist research and one that has remained largely stable in its definition over time. Rather than requiring justification, its inclusion here serves to emphasise its importance for realist inquiry, as it underpins the process of theoretical generalisation.
Retroduction	*Mode of inference used in realist research to identify and theorise the underlying, hidden mechanisms that generate observed events by reasoning backwards from empirical patterns to the conditions and structures that must exist for those events to occur*	Mukumbang [63]; Jagosh [64]	The concept of retroduction has evolved over time, with key contributions from Peirce [65], Bhaskar [66] and Danermark et al. [67]. The definition adopted here reflects some of the most recent and applied understanding of this mode of inference.
Generative causation	*Model of causality which postulates that outcomes are produced by underlying mechanisms operating within specific contexts. Generative causation explains how and why an intervention brings about change.*	Pawson and Tilley [49]	Although the notion of generative causation has been widely debated, this review adopts the definition proposed by Pawson and Tilley [49], whose work places particular emphasis on generative causation as the core of realist explanation.
Thrive	*Outcomes in which service user researchers can contribute their expertise effectively and sustainably within academic mental health research settings. Thriving is characterised by meaningful participation, recognition of expertise, and the ability to develop and maintain a productive role over time. It does not imply uninterrupted success or absence of challenge, but reflects positive trajectories in SURs’ experiences, contributions, and role sustainability.*	Lovell-Norton et al. [68]	Used as an analytical heuristic to support examination of what works, for whom, and in what contexts. Thriving does not imply constant or idealised success, but reflects trajectories characterised by participation, recognition, and influence across a continuum of experiences. Choice of wording inspired by the chapter of Lovell-Norton et al. [68] and validated through stakeholder consultation.
Survive	*Outcomes in which service user researchers’ contributions are constrained or marginalised, limiting the extent to which their expertise can be effectively or sustainably enacted within academic mental health research settings. Survival may be reflected in restricted roles, under-recognition of expertise, reduced influence, or continued participation despite significant difficulty, and may include trajectories of withdrawal, disengagement, or exit.*	Lovell-Norton et al. [68]	Used as an analytical heuristic to support examination of what works, for whom, and in what contexts. Survival reflects a continuum of experiences characterised by restricted participation, reduced influence, or persistence in roles despite ongoing difficulty, and may include trajectories of disengagement or exit. Choice of wording inspired by the chapter of Lovell-Norton et al. [68] and validated through stakeholder consultation.

To initially focus the review, an exploratory, non-systematic search of the literature was conducted in May 2025, followed by an update search in September 2025 to capture newly published material. This approach was informed by the exploratory strategy described by Gupta et al. [3]. Searches were conducted in Google Scholar, PubMed, and MEDLINE to identify conceptually rich papers addressing organisational and academic team dynamics, SUR contributions, intended outcomes of these roles, and the potential contexts and mechanisms at play. These databases were selected as they provided broad multidisciplinary coverage alongside structured biomedical indexing. Together, these databases enabled rapid mapping of relevant literature, identification of key terminology and refinement of the review focus prior to developing a formal search strategy. The searches yielded 2,828 records across all databases. All identified titles were screened by title and abstract, with 50 papers flagged as potentially contributing to theory development. These 50 papers were assessed to capture a broad conceptual understanding of the topic. Papers were assessed for their relevance and conceptual richness, particularly their potential to inform understanding of organisational and academic team dynamics, SUR roles and contributions, intended outcomes and relevant contextual conditions and mechanisms. 15 papers were then purposively selected for in-depth review because they provided the most theoretically relevant and detailed evidence for developing the IPT. This sample was considered sufficient for an exploratory phase focused on theory development rather than comprehensive literature coverage, described in detail in Step 2. The characteristics and key findings of the 15 papers are presented in Table 2.

**Table 2:** Characteristics of included studies for initial programme theory development stage

Authors	Study title	Year	Setting	Type of source	Aim of study	Key findings
Beames et al. [2]	A New Normal: Integrating Lived Experience Into Scientific Data Syntheses	2021	Mental health and clinical research broadly	Peer-reviewed opinion/perspective.	To identify the limited integration of lived-experience perspectives into scientific evidence syntheses and make the case for appropriately involving people with lived experience in future reviews and meta-analyses	The authors argue that integrating lived experience throughout evidence synthesis could improve research relevance, quality, practical impact, knowledge translation and the development of interventions that reflect users’ needs and preferences.
Davis et al. [6]	Reporting lived experience work	2024	Mental health research and scientific publishing	Peer-reviewed comment/editorial.	To explain the importance of involving people with lived experience in mental health research and announce clearer, more systematic reporting requirements	The authors argue that lived-experience involvement can improve research quality, relevance, feasibility, implementation, ethics, trust and knowledge translation
Dembele et al. [69]	Researching With Lived Experience: A Shared Critical Reflection Between Co-Researchers	2024	Mental health academic research	Peer-reviewed qualitative research article	To identify barriers and facilitators to meaningful participation by lived-experience co-researchers.	Seven themes were identified: (1) ethics as an ongoing process; (2) recruitment and diversity of co-researchers; (3) creating safety for all; (4) supporting flexible ways of partnering; (5)capacity building and training; (6) strengths-based positioning; and (7) addressing power imbalances.
Gupta et al. [3]	Understanding the identity of lived experience researchers and providers: A conceptual framework and systematic narrative review	2023	Mental health academic research	Peer-reviewed systematic narrative review	To examine how the identities of lived-experience researchers and providers are conceptualised and to develop a framework describing how these roles influence identity.	Five identity positions were found: professional, service user, integrated, unintegrated, and liminal. The authors developed the EMERGES framework comprising seven influences on identity
Hawke et al. [70]	Embedding lived experience into mental health academic research organizations: Critical reflections	2022	Mental health academic research	Peer-reviewed viewpoint article/critical reflection	To identify key issues that research institutions should consider when developing initiatives that recognise, support and use the lived experience of academic mental health researchers	The authors identify several considerations: how lived experience is defined; who qualifies as a lived-experience academic researcher; the objectives of institutional initiatives; how researchers balance academic and lived-experience identities; what roles they should hold; how many researchers should be involved; and how they should be recruited or supported
Hogg et al. [38]	‘Lemons to lemonade’: Identity integration in researchers with lived experience of psychosis	2025	Mental health academic and health research	Peer-reviewed qualitative research	To explore how researchers with lived experience of psychosis develop social identities and integrate their lived-experience identity with their researcher or academic identity	Two overarching categories were identified: (1) the basis for social identification and (2) the complexity of identity integration within academia.
Lynch et al. [71]	Credit where it’s due: Recognising lived experience in research authorship	2025	Mental health academic research	Peer-reviewed cross-sectional survey research	To identify current practices and preferences regarding whether and how authors’ lived experience should be acknowledged when they contribute lived-experience expertise to health and healthcare research outputs.	Most respondents believed authors’ lived experience should be publicly recognised: 63.9% of lived-experience respondents and 70% of academic researchers supported recognition in all or most outputs
Okoroji et al. [22]	Epistemic injustice and mental health research: A pragmatic approach to working with lived experience expertise	2023	Mental health research	Peer-reviewed perspective	To examine how epistemic injustice operates in mental health research and propose practical principles for progressing towards epistemic justice.	The authors identify two major barriers: (1) elite capture, where a small and relatively privileged group of lived-experience contributors is selected because they can conform to academic norms; and (2) epistemic exploitation, where marginalised people are repeatedly expected to provide testimony about oppression without having meaningful influence over knowledge production
Omeni et al. [23]	Service user involvement: Impact and participation: A survey of service user and staff perspectives	2014	Community mental health services	Peer-reviewed cross-sectional mixed-methods survey	To examine levels of participation in service-user involvement among mental health service users and frontline professionals	45.7% of service users and 55.9% of professionals had participated in at least one involvement activity. Both groups generally perceived service-user involvement positively, particularly in supporting decision-making, improving services, increasing choice and control, enhancing self-esteem and recovery, and helping service users feel heard
Richmond et al. [72]	Creating positive experiences of involvement in mental health research	2023	Mental health academic research	Peer-reviewed personal view	To explore how involvement in mental health research affects people who contribute lived-experience expertise	Six characteristics of positive involvement were identified: (1)reframing painful memories, (2) recognising value, (3) practising reciprocity, (4) bridging gaps, (5) countering stigma, and (6) challenging established narratives.
Rose [73]	Collaborative research between users and professionals: Peaks and pitfalls	2003	Mental health academic research	Peer-reviewed opinion and debate	To provide an overview of collaborative research between mental health service users and clinical academics, illustrate different forms and potential benefits of collaboration, identify obstacles, and suggest future directions.	The article distinguishes consultative, collaborative and user-led research, with user-led research involving service users controlling all stages of the research process. The author recommends building research capacity among service users and academics, offering training and career development and demonstrating that collaborative research produces rigorous and useful evidence.
Rose et al. [74]	A model for developing outcome measures from the perspectives of mental health service users	2011	Mental health academic research	Peer-reviewed methodological/model-development	To describe the SURE model for developing robust outcome measures entirely from the perspectives of mental health service users	Across applications of the model, the resulting measures demonstrated robust psychometric properties and identified priorities sometimes absent from conventional measures, including peer support, information provided directly to service users, safety and quality of staff interactions
Samra [75]	Conceptualising lived experience in mental health research: Problems, insights and implications	2025	Mental health research and practice broadly	Peer-reviewed conceptual review	To examine limitations in conceptualising mental health lived experience mainly through epistemic concerns and to argue for greater attention to the ontological domain	The author argues that an emphasis on lived experiencers as credible “knowers” can overlook how serious mental distress may disrupt sensory perception, self-understanding, decision-making and trust in one’s sense of reality. Psychiatric categorisation and diagnostic labels can create ontological harm or violence by defining a person’s reality as false.
Speyer & Ustrup [15]	Embracing dissensus in lived experience research: The power of conflicting experiential knowledge	2025	Mental health academic research	Peer-reviewed personal view/conceptual reflection	To examine how conflicting lived-experience perspectives can be handled in mental health research and to present an approach that treats disagreement as a potential source of deeper understanding	The authors propose a two-step model: first, identify the genuine point of disagreement or “true negation”; second, clarify the epistemic goal of the inquiry. Consensus may be useful when a generalised answer is required, but dissensus should be preserved when the aim is innovation
Staley et al. [76]	Service users as collaborators in mental health research: Less stick, more carrot	2013	Mental health academic research	Peer-reviewed editorial article incorporating a small-scale qualitative evaluation	To examine how service users were involved in Mental Health Research Network-supported studies, assess their influence on research, identify challenges faced by researchers, and consider how organisations could encourage more meaningful involvement.	Service-user involvement influenced study design in 61% of evaluated projects, including research questions, outcome measures, interventions, recruitment materials and practical arrangements. More intensive involvement as co-researchers or research-team members had broader effects, improving relevance, recruitment, retention, interview quality and interpretation.

The next stage involved a concept mining exercise to identify key ideas and recurring themes across the selected literature. Concept mining refers to the systematic identification, extraction and interpretation of theoretical constructs and explanatory concepts from diverse sources to inform programme theory development [56]. Through this process, and in discussion with the project team, several core concepts were identified as central to the emerging programme theory, including epistemic justice and institutional dynamics.

Each selected paper was then read in full to identify passages suggesting plausible CMO configurations (CMOCs). Initial explanatory “hunches” were first recorded manually through research journaling to support reflective and open-ended engagement with the material and subsequently transcribed into a digital document for refinement. During transcription, CMOCs were iteratively refined to clarify relationships between contexts, mechanisms and outcomes. While contextual factors were often explicitly described in the literature, mechanisms frequently required inference. To identify these underlying causal processes, retroductive reasoning was employed, an explanatory inference approach well suited to uncovering hidden mechanisms that generate observable outcomes [63, 64].

Following identification, CMOCs were organised into two broad categories: those suggesting organisational and team dynamics associated with outcomes in which SURs thrive, and those associated with outcomes in which SURs survive [68]. To further refine the analysis, CMOCs were transferred into an Excel spreadsheet and examined against three criteria: their linkage to concepts identified through concept mining, their relevance to understanding the dynamic relationship between organisational and team contexts and SUR contributions and their prominence within the literature.

In parallel, engagement with substantive theory was a critical step in refining the IPT beyond engagement with the literature [77]. Shearn et al. [78] caution against relying solely on empirical data to develop IPTs, noting risks such as confirmation bias and the generation of multiple, unconnected theories. To mitigate these risks and in line with RAMESES quality standards, substantive theory was introduced to prioritise CMOCs with the greatest explanatory potential. This reduced the risk of developing numerous disconnected propositions based solely on empirical findings. Candidate theories were identified through a rapid, adapted BeHEMoTh search of PubMed and Google Scholar [79], followed by iterative searching and discussion with the wider team. After the appraisal of potentially relevant theories, the Institutional Logics Perspective (ILP) [80] was selected as the theory most relevant to the developing IPT. The ILP proposes that individual and organisational behaviour is shaped by institutional logics: systems of values, assumptions, rules, and practices that influence how people understand and respond to organisational expectations. Within HEMHRCs, professional, bureaucratic, market, and community logics may coexist, compete or combine, shaping how SUR roles are valued and supported. The ILP therefore provided a field-specific realist framework for examining how institutional conditions interact with individual agency to activate mechanisms that enable SURs to thrive or require them merely to survive [81, 82]. Its attention to macro, meso, and micro-level logics also supported the development of an IPT that embraced complexity by considering the relationships between wider institutional dynamics and SURs’ individual experiences [83].

Once the substantive theory was selected, each CMOC was assessed for its alignment with the core principles of the ILP using a three-point rating scale (1 = low alignment; 2 = moderate alignment; 3 = strong alignment). This assessment captured the extent to which each CMOC reflected interactions between institutional logics, agency and organisational outcomes. CMOCs rated as demonstrating strong alignment were prioritised for inclusion in the preliminary IPT due to their theoretical coherence and explanatory potential.

Stakeholder consultation then provided critical feedback on the preliminary IPT, supporting its refinement [84, 85]. This constituted the first of three planned consultation stages and involved purposively sampled participants with expertise in SUR roles, including SURs currently working in universities, coordinators of lived experience research, former SURs, and academics working alongside people with lived experience [86]. The consultation took the form of a 90-minute Microsoft Teams meeting, supplemented by an individual 60-minute meeting for one participant unable to attend the original meeting. The session included an overview of the realist approach and an accessible presentation of the IPT, followed by discussion of the research questions and initial theoretical assumptions. Stakeholders acted as theory informants by identifying assumptions within the preliminary IPT that should be confirmed, challenged, expanded, or revised. Their feedback and the resulting changes were recorded in a live “you said, we did” document for transparency and accountability, with further details on recruitment and involvement provided in the Stakeholder Involvement section.

**Fig. 1:**
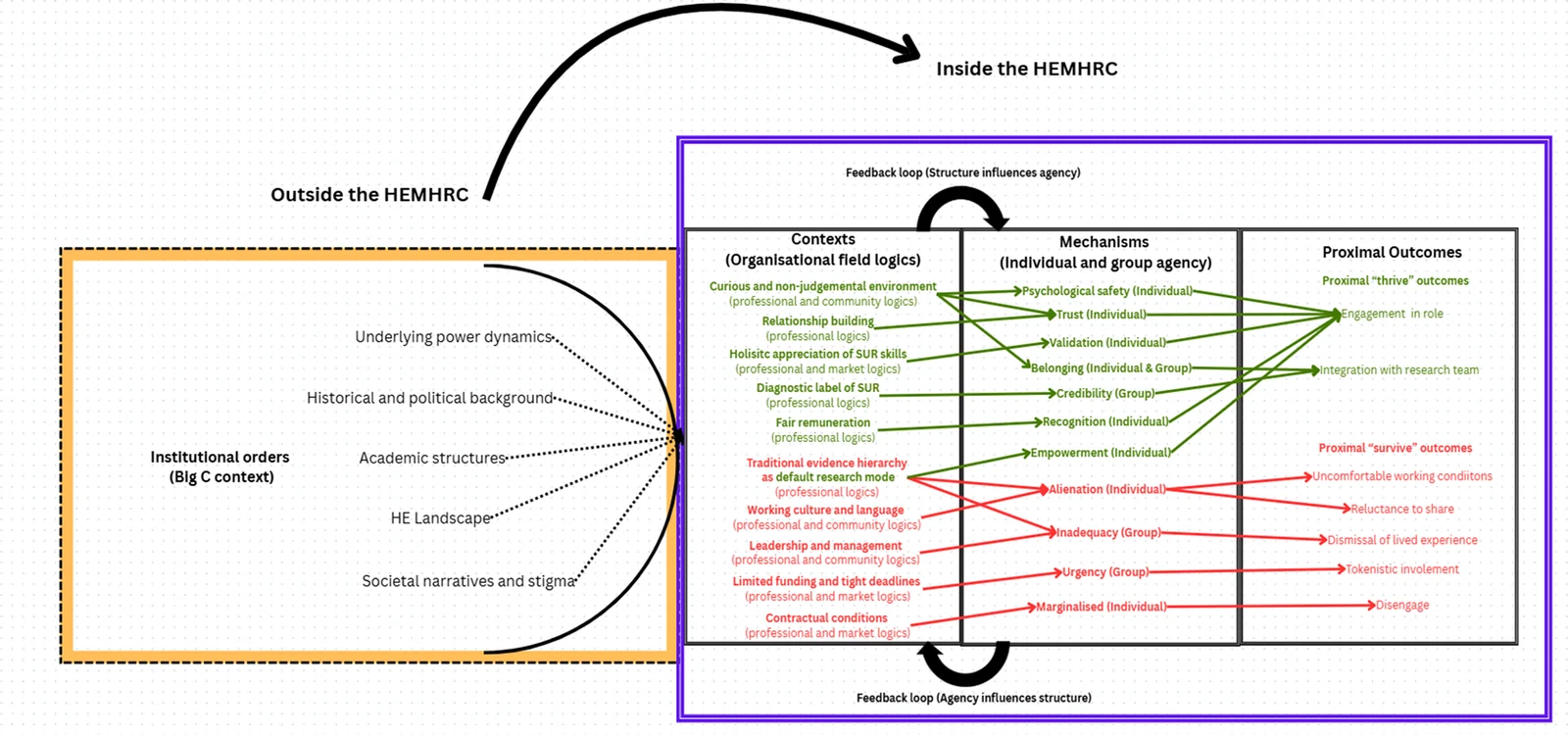
Initial rough programme theory visualisation diagram. Legend: This figure illustrates the IPT developed during the scoping phase of the realist review. The diagram depicts how institutional orders external to HEMHRCs shape organisational and team contexts, which in turn trigger mechanisms influencing SURs’ experiences and contributions. Green pathways represent mechanisms associated with proximal “thrive” outcomes, while red pathways represent mechanisms associated with proximal “survive” outcomes. Bidirectional arrows indicate feedback loops between agency and structure. The diagram includes 13 CMOCs that informed the subsequent search strategy and analytic phases of the review. Abbreviations: HEMHRC, Higher Education Mental Health Research Centre; SUR, Service User Researcher; HE, Higher Education; Big C context, overarching contextual conditions external to the organisation that shape institutional logics operating within the system

Together, these processes sharpened the focus of the IPT and guided the review towards a more specific line of inquiry, centred on organisational, team, and individual dynamics and their reciprocal relationship with SUR experiences and contributions. Following incorporation of stakeholder feedback, a refined IPT visual was developed via Canva with 13 CMOCs to guide the subsequent search strategy and stages of the realist review (see Fig. 1).

### Example tentative CMO

When an academic organisation offers SURs remuneration, employment conditions and benefits comparable to other team members (C), SURs are more engaged and able to focus fully on contributing to projects (O) because they feel recognised and equal, reducing preoccupation with systemic workplace injustices (M).

**Step 2: Searching for Evidence**

Building on prior research examining SUR roles in higher education, this review will search MEDLINE, PsycINFO, CINAHL, Scopus, and Web of Science to identify relevant literature [3]. An initial free-text search strategy will be developed in collaboration with an information specialist, using Boolean logic, proximity operators and keyword fields to capture relevant documents (see Additional File 2 for an example search strategy). To ensure comprehensive coverage, database searches will be supplemented with targeted grey literature searches in Google, ProQuest Dissertations & Theses Global, NHS/NIHR Evidence and Journals Library, Overton, Policy Commons and targeted searches of relevant organisational and funder websites and citation tracking, in line with recommendations for realist reviews [54].

An iterative search process will be employed to identify, test, and refine emerging programme theories through the progressive inclusion of new evidence. This process will be responsive to insights generated from the literature and evolving contextual understanding as the review progresses [51, 87]. Following each completed search and analytical cycle, the emerging programme theories will be assessed for theoretical saturation; where gaps or uncertainties remain within particular contexts, mechanisms, or outcomes, targeted supplementary searches will be undertaken, and any additional evidence will be appraised and incorporated using the same review and synthesis procedures. In addition to studies focusing explicitly on SUR roles, the search will include evidence from comparable roles and interventions in other academic or research settings where similar mechanisms are likely to operate, supporting further refinement and strengthening of the programme theory [77].

**Step 3: Selection and Appraisal of Studies**

Documents retrieved from database and grey literature searches will be imported into Rayyan to support initial deduplication and screening. During title and abstract screening, the inclusion criteria will be intentionally broad to capture conceptually useful “nuggets” relevant to programme theory development [88]. One team member (A.A.B.) will conduct the initial screening, consulting with other members of the research team (F.L., A.H.) as needed, with disagreements resolved through discussion and consensus. To strengthen the rigour of screening decisions, a second reviewer will independently screen 20% of records. Full-text screening will follow the same procedures, with one reviewer (A.A.B.) conducting the assessment in ongoing consultation with the research team (F.L., A.H.), and an independent second reviewer screening 20% of full-text articles to ensure consistency and rigour.

To be included, documents must address organisational and/or team dynamics within HEMHRCs and examine how these influence, and are influenced by, SURs’ contributions to, and experiences of, mental health research. In this review, organisational and/or team dynamics refer to the formal and informal structures, processes, relationships, and cultural norms within academic research settings that shape how work is organised, decisions are made and roles are enacted (e.g. leadership practices, governance arrangements, power relations, communication, and support structures). This does not include individual-level characteristics or interpersonal experiences in isolation, unless they are explicitly linked to wider organisational or team-level processes or contexts. Documents published from 2002 onwards will be included, as this marks the first documented SUR role at the University of Melbourne [89]. To be included, SURs must hold a substantive contract (which could be casual, part-time or full time) with a minimum of two hours per week allocated to a research project, which may be across one or multiple projects within a HEMHRC. This criterion allows inclusion of the wide range of ways in which SUR roles are enacted, while ensuring a minimum level of engagement for individuals to meaningfully interact with, and be shaped by, organisational and team dynamics over time [27]. Eligibility is limited to roles recruited into academic settings, as confirmed through the role descriptions and definitions provided in the included documents. Studies involving staff who happen to have lived experience but were not recruited on this basis will be excluded. Where the purpose or nature of the role is unclear, authors will be contacted for clarification. Empirical studies using quantitative, qualitative or mixed-methods designs will be eligible, consistent with realist review principles that draw on diverse evidence types to inform theory development [54]. Grey literature (e.g., university strategies for lived experience integration, theses, and relevant reports) will also be considered. Included documents must provide outcome-relevant data relating to SURs’ and other key stakeholders’ experiences and/or contributions and indicate how these are facilitated or constrained by contextual features of academic systems. Inclusion criteria will be refined iteratively as the review progresses to ensure the selection of the most relevant and conceptually rich evidence for developing and refining IPTs, and to enable further focusing if the volume of records is high [50, 90].

Quality appraisal will follow RAMESES principles of relevance, richness and rigour [54], using a three-point Likert scale to assess each source’s contribution to programme theory development, consistent with Dada et al. [91]. Relevance will assess the extent to which a source contributes to refining, supporting, or challenging the IPT; richness will capture the depth and explanatory detail provided; and rigour will be considered at both the data level (e.g., credibility and trustworthiness) and the theory level (e.g., coherence and plausibility of resulting CMOCs). All assessments will be documented transparently. Lower-rigour sources will not be excluded automatically and will be considered for their potential contribution of theoretically useful “nuggets” [88].

**Step 4: Data Extraction**

Data extraction will be conducted in accordance with previous realist reviews [92, 93]. A bespoke data extraction form will be developed to support programme theory development and refinement, rather than to summarise study findings alone (see Additional File 3). Data will be extracted with explicit attention to how each segment of evidence contributes to the development, testing, or refinement of CMOCs, allowing emerging insights to iteratively shape the programme theory. Key study characteristics will be recorded in an Excel spreadsheet to support transparency and synthesis across sources. Extracted characteristics will include authors and year of publication, study design, setting (including country or countries), population, role components, stage of implementation (e.g. exploratory, pilot, or established practice), reported outcomes, and any identified contributions towards programme theory development via contextual conditions and mechanisms.

**Step 5: Synthesis and Refinement of Theories via Analysis and Stakeholder Consultation**

The synthesis stage will apply a realist logic of analysis to iteratively develop and refine the IPT alongside ongoing data extraction, resulting in progressively refined CMOCs [77]. Analysis will be guided by retroductive reasoning to generate causal explanations, focusing on how and why particular mechanisms are triggered (or not) within specific organisational and team contexts [63, 64].

Data will be analysed using the analytic tools of coding, consolidation and conceptual mapping [94, 95]. All included peer reviewed and grey literature sources will be imported into NVivo and coded using realist principles to identify potential CMOCs, with annotations used to document interpretive decisions and retroductive inferences [96]. Conceptual codes will be applied to capture key elements and emerging patterns, working backwards from observed outcomes to infer the mechanisms likely generating these outcomes, as well as the contextual conditions shaping their activation. The coded sources will then be consolidated and mapped against the developing CMOCs to determine whether it confirms, refutes, or refines the IPT. Following each analytical cycle, convergence and gaps within the evidence will be examined to assess whether further targeted searching is required and whether theoretical saturation has been reached by asking (1) whether the evidence fits within the boundaries of the IPT and (2) whether it strengthens explanatory power and helps explain generative causation through emerging patterns.

Identified CMOCs will then be extracted into Microsoft Excel to support consolidation, enabling configurations to be compared, grouped and iteratively refined while maintaining a transparent audit trail of analytic decisions. Conceptual mapping will be conducted using Canva, where visual representations will be developed to illustrate relationships between CMOCs and to support abstraction and theory refinement. To enhance rigour, audit documents will be maintained in Microsoft Word, compiling annotated data extracts and conceptual maps at key stages of synthesis. This documentation will support transparency and strengthen interpretive validity and ontological plausibility.

Finally, the evolving IPT, framed at a middle-range level of abstraction, will be further refined through engagement with evidence from the included literature and relevant substantive theory. The IPT provides a provisional, study-specific account of how contextual conditions may trigger mechanisms that enable SUR to thrive or merely survive. It will guide data collection and analysis and will be iteratively tested and refined against the evidence. The refined middle-range programme theory will offer a more developed and transferable explanation of how, why, for whom and under what conditions these outcomes occur, while remaining sufficiently specific to be empirically investigated [60]. Theory refinement will involve processes of juxtaposing, adjudicating, reconciling, consolidating, and situating evidence, consistent with realist review principles [50]. Emerging CMOCs and theoretical refinements will be reviewed in collaboration with the research team and key stakeholders as a distinct stage, during which stakeholders will be invited to confirm, extend, challenge or refine the revised IPT.

### Stakeholder involvement

Stakeholder consultation is integral to realist reviews, with stakeholders playing an ongoing role in refining IPTs. Involving key stakeholders helps ensure that the research addresses real-world concerns, generates meaningful and applicable findings and strengthens programme theory development by deliberately incorporating the perspectives of those with direct experience of the phenomena under study [97, 98]. In this project, stakeholders are involved from the outset to support the development and refinement of IPTs, offering insights into mechanisms and outcomes that operate across different academic contexts [99].

Consistent with realist principles, stakeholders were purposively recruited for their potential to contribute relevant experiential and professional knowledge to programme theory development and refinement [86]. University profiles were reviewed to identify individuals with experience either as SUR or as academics working alongside SUR across a range of higher education institutions. Potential participants were contacted by email, and seven of those approached agreed to take part, representing a diverse range of roles and perspectives related to SUR involvement in academic mental health research. All stakeholders were contacted prior to the review to assess interest, relevance, project fit, and availability. Each participant completed an expression of interest form and is remunerated at a rate of £35 per hour for their contributions. Stakeholder consultations are conducted synchronously via Microsoft Teams and organised via Doodle, alongside a shared Google Document that enables asynchronous input. This document serves as a central space to collate feedback, track progress and document decisions, supporting transparency and accountability. It also provides a record of workshop processes and outcomes to promote trust and shared ownership of the review.

Three stakeholder meetings are planned across the review. Each meeting is preceded by a clear agenda and objectives, and guided by principles of open dialogue and critical engagement. Stakeholders are encouraged to share experiences and insights that align with or challenge emerging theories, including perspectives not directly reflected in the literature. Discussions are recorded and transcribed to support transparency and in-depth analysis, with realist analysis logic principles applied to refine programme theories. The first stakeholder meeting has already taken place and is described in Step 1 above. This session introduced the project and realist approach and explored early IPTs. Participants were provided in advance with a project summary, agenda and supporting materials. Verbal informed consent was obtained from all stakeholders at the start of the first session for the sessions to be recorded and transcribed for the purposes of theory building and transparency, with further informed consent to be sought at the beginning of each subsequent scheduled meeting.

The second meeting will occur midway through the review to reflect on the selected literature and identify gaps, including relevant grey literature not captured through formal searches. The third meeting will focus on discussing refined CMOCs and emerging theoretical insights, with stakeholder feedback incorporated to further strengthen and ground the final programme theory in real-world academic contexts beyond the published literature. Stakeholder contributions will be acknowledged in dissemination and knowledge mobilisation materials, and stakeholders will be invited to co-author relevant project outputs where appropriate.

### Dissemination plans

This research will generate targeted outputs designed to maximise impact by improving both the contributions and experiences of SURs within HEMHRCs and organisational and team dynamics. Dissemination activities will include a final peer-reviewed publication, alongside presentations at academic and applied conferences to share emerging findings and engage in critical dialogue. Findings will also be disseminated through the research team’s professional networks and social media platforms to reach a wider and more diverse audience. To ensure accessibility and relevance across audiences, stakeholders will be consulted throughout to shape outputs that are tailored to the needs of key actors, including SURs, academic staff, and institutional leaders [100].

In addition to academic outputs, accessible guidance documents will be co-developed with stakeholders to provide clear, practical recommendations for designing and sustaining SUR roles within mental health research. These resources will be tailored for university leaders, research managers and SURs. Lay summaries and infographics will also be produced to communicate how, for whom, and in what contexts SURs are most likely to contribute meaningfully, and which organisational conditions enable or constrain this. Through this multi-pronged dissemination strategy, the study aims to ensure that findings extend beyond academic publication to inform policy and practice, shaping how academic organisational dynamics influence, and are influenced by, SURs’ experiences and contributions.

## Discussion

Understanding the contexts and mechanisms that shape SURs’ experiences and contributions is essential for explaining how, why, and under what circumstances these roles can be successfully embedded within academic research environments. Without such understanding, attempts to support and expand SUR involvement risk being based on assumptions rather than evidence, potentially limiting the sustainability and impact of these roles. Making these mechanisms and underlying theories explicit seeks to strengthen organisational and team dynamics within HEMHRCs, while also improving the experiences and contributions of SURs to support better working environments, role design and mental health research outcomes. By developing a programme theory that brings to the surface the often hidden but generative causal forces shaping practice, the review aims to ensure that organisational and team contexts enable SURs to thrive rather than merely survive, allowing them to focus on what matters most: making meaningful impact by drawing on their full skillsets and expertise to advance mental health research.

The iterative steps outlined above will guide the review from the completed phases of scope refinement and IPT development through systematic and grey literature searching, quality appraisal using realist standards of relevance, richness, and rigour, CMOC development through realist analytical logic and sustained stakeholder involvement throughout. Together, these processes will ensure that the findings are meaningful and impactful for all key stakeholders engaged in this area.

## Electronic supplementary material

Below is the link to the electronic supplementary material.

Supplementary Material 1

Supplementary Material 2

Supplementary Material 3

## Data Availability

No datasets were generated or analysed during the current study.

## Abbreviations

C: Context
CMO: Context-Mechanism-Outcome
CMOC: Context-Mechanism-Outcome Configuration
HEMHRC: Higher Education Mental Health Research Centre
ILP: Institutional Logics Perspective
IPT: Initial programme theory
LENS: Lived experience network of scholars
M: Mechanism
O: Outcome
RAMESES: Realist and meta-narrative evidence syntheses: evolving standards
SUR: Service user researcher
SURE: Service User Research Enterprise
