# Supplementary material for: How, why, for whom, and in what contexts organisational and team dynamics shape, and are shaped by, service user researchers in higher education mental health research centres: a realist review protocol
Source: Res Involv Engagem. 2026 Sep 23;12:154. doi: 10.1186/s40900-026-00980-8 (PMC13599209; doi:10.1186/s40900-026-00980-8)
Supplement: Supplementary file 2 [file 40900_2026_980_MOESM2_ESM.docx]

**Additional File 2. Search Strategy**

**Academic Databases – Medline (via EBSCO) Example (MeSH, Title (Ti), and Abstract (Ab) fields)**

| **Search** | **Search terms** |
| --- | --- |
| **S1** | XB ((("lived experience*" OR "service user*" OR survivor* OR user-led OR patient* OR public OR customer* OR peer OR "participatory action") N5 (research* OR expert* OR involvement)) OR PPI OR PPIE OR ((public OR "service user*" OR consumer*) N5 (involvement)) OR (( "lived experience") N5 (advisor* OR consultant* OR academic* OR practitioner*)) OR "research partner" OR co-researcher* OR "co researcher*" OR coresearcher* OR co-production OR "co production" OR "participatory research*")) |
| **S2** | (MH "Universities") |
| **S3** | (MH "Education, Graduate") |
| **S4** | (MH "Academia") |
| **S5** | S2 OR S3 OR S4 |
| **S6** | XB ((higher OR tertiary) N3 (educat* OR postsecond* OR academia)) OR ((academic) N3 (setting* OR educat* OR institution* OR research)) OR ((teaching) N3 (“research institution*" OR universit* OR college*)) OR (("academic) N3 (institution* OR campus* OR facult*)) OR ((higher) N3 (education OR institution* OR HEI)) OR ((academic) N3 (environment* OR workplace*)) OR ((university) N3 (based OR research OR centre*)) OR ((research) N3 (center* OR "university* OR research OR group*)) OR ((academic) N3 (“research setting*)) OR ((academic) N3 (department*)) OR ((UK) N3 ((universit*)) OR ((UK) N3 (higher OR educat*)) OR ((HE) N3 (sector)) OR ((HE) N3 (institution*)) |
| **S7** | S5 OR S6 |
| **S8** | (MH "Mental Health") OR (MH "Mental Health Recovery") |
| **S9** | XB ((mental) N3 (health* OR illness OR distress OR Psychiat*)) OR ((mental) N3 (health service*)) OR ((psycholog*) N3 (wellbeing OR recover*)) OR ((mental) N3 (health recover*)) OR ((mental) N3 (“health care" OR healthcare)) OR "mental health condition*" OR ((psychiatric) N3 (survivor*)) OR ((“mental health”) N3 (lived experience*")) |
| **S10** | S8 OR S9 |
| **S11** | XB (("impact*" OR "effect*" OR "influenc*" OR "chang*" OR "transform*" OR "outcome*" OR "consequence*" OR "benefit*" OR "result*" OR "evidence of impact*" OR "measurable outcome*" OR "difference made" OR "added value" OR "value added" OR "legacy" OR "improv*" OR "innov*" OR "shift*" OR "progress*" OR "contribut*" OR "success*" OR "effectiv*" OR "evaluat*" OR "lesson* learn*" OR "advanc*" OR "develop*" OR "reform*" OR "enhanc*" OR "change in culture" OR "institutional change" OR "policy change" OR "practice change" OR "cultural shift" OR "organi?ational change" OR "positive impact*" OR "negative impact*" OR "inadequ*" OR "feedback" OR "outcome measure*" OR "performance indicator*" OR "organi?ational culture" OR "organi?ational climate" OR "workplace culture" OR "academic culture" OR "team climate" OR "team dynamic*" OR "power dynamic*" OR "epistemic injustice" OR "epistemic authority" OR legitim* OR tokenis* OR "meaningful involvement" OR "meaningful engagement" OR "inclusion" OR "inclusive" OR "exclusion" OR "excluded" OR "belonging" OR "marginalis*" OR "alienat*" OR "stigma" OR stigmati?* OR "patronising" OR "psychological safety" OR "safe space*" OR "trust" OR "trusting" OR "reluctant" OR "role clarity" OR "role conflict" OR "role ambiguity" OR "credibl*" OR "boundary*" OR "team cohesion" OR "collaboration" OR "collaborative" OR "partnership" OR "partnership working" OR "integrat*" OR "experience*" OR "contribution*" OR "engagement" OR "participation" OR "research engagement" OR "research participation" OR "validation" OR "validated" OR "having a voice" OR "being heard" OR "pay" OR "paid" OR "unpaid" OR "remuneration" OR "equal pay" OR "wage*" OR "salar*" OR "hourly rate" OR "living wage" OR "working condition*" OR "employment condition*" OR "contract*" OR "casual*" OR "precarious employment" OR "precarity" OR "job security" OR "workplace inequality" OR "fair pay" OR "fair remuneration" OR "employment benefits" OR "staff benefits" OR "urgen*" OR "empower*" OR "recogni*" OR "dismiss*" OR "purpose*" OR leadership*" OR "supervision")) |
| **S12** | S1 AND S7 AND S10 AND S11 |

**Grey Literature – Example**

| **Search** | **Search terms** |
| --- | --- |
| **S1** | "lived experience" OR (researcher* OR academic* OR "research partner*" OR "co-researcher*" OR  "consumer academic*" OR "peer researcher*" OR "service user researcher*") AND  (university OR "higher education" OR "HE institution" OR "research centre" OR "research center" OR  "academic department" OR "academic workplace" OR "research group") AND ("mental health" OR psychiatry OR "mental illness" OR "mental distress" OR "mental health research") AND  ("co-production" OR "coproduction" OR "co-produced" OR "meaningful involvement" OR tokenism OR  "tokenistic" OR "organi?ational culture" OR "workplace culture" OR  "academic culture" OR "psychological safety" OR "power dynamics" OR "power imbalance" OR  "working conditions" OR "precarious employment" OR precarity OR "casuali?ation" OR "equal pay" OR remuneration OR "fair pay" OR "lived experience leadership" OR "leadership*" OR "supervision" OR "urgen*" OR "empower*" OR "recogni*" OR "dismiss*" OR "purpose*") |
