# Supplementary material for: How, why, for whom, and in what contexts organisational and team dynamics shape, and are shaped by, service user researchers in higher education mental health research centres: a realist review protocol
Source: Res Involv Engagem. 2026 Sep 23;12:154. doi: 10.1186/s40900-026-00980-8 (PMC13599209; doi:10.1186/s40900-026-00980-8)
Supplement: Supplementary file 3 [file 40900_2026_980_MOESM3_ESM.docx]

**Additional File 3. Data extraction form.**

| Number | Author | Year | Country | Setting | Study Design | Participant Characteristics | Aim of study | Role components | Implementation stage | Reported outcomes | Key findings | Contribution to programme theory development | Alignment with substantive theory |
| --- | --- | --- | --- | --- | --- | --- | --- | --- | --- | --- | --- | --- | --- |
|  |  |  |  |  |  |  |  |  |  |  |  |  |  |
